# Ultrasound-Assisted Nanoemulsion Loaded with Optimized Antibacterial Essential Oil Blend: A New Approach against *Escherichia coli*, *Staphylococcus aureus*, and *Salmonella* Enteritidis in Trout (*Oncorhynchus mykiss*) Fillets

**DOI:** 10.3390/foods13101569

**Published:** 2024-05-17

**Authors:** Luiz Torres Neto, Maria Lucia Guerra Monteiro, Bruno Dutra da Silva, Maxsueli Aparecida Moura Machado, Yhan da Silva Mutz, Carlos Adam Conte-Junior

**Affiliations:** 1 Technological Development Support Laboratory (LADETEC), Center for Food Analysis (NAL), Cidade Universitária, Rio de Janeiro 21941-598, RJ, Brazil; mariaguerra@id.uff.br (M.L.G.M.); brunodutrads@gmail.com (B.D.d.S.); maxsuelii@ufrj.br (M.A.M.M.); yhanmutz@ufrj.br (Y.d.S.M.); conte@iq.ufrj.br (C.A.C.-J.); 2 Graduate Program in Food Science (PPGCAL), Institute of Chemistry (IQ), Federal University of Rio de Janeiro (UFRJ), Cidade Universitária, Rio de Janeiro 21941-909, RJ, Brazil; 3 Graduate Program in Veterinary Hygiene (PPGHV), Faculty of Veterinary Medicine, Fluminense Federal University (UFF), Vital Brazil Filho, Niterói 24220-000, RJ, Brazil; 4 Graduate Program in Sanitary Surveillance (PPGVS), National Institute of Health Quality Control (INCQS), Oswaldo Cruz Foundation (FIOCRUZ), Rio de Janeiro 21040-900, RJ, Brazil

**Keywords:** volatile oils, natural compounds, synergy, pathogen, storage, desirability function

## Abstract

This study aimed to obtain and characterize an oil-in-water nanoemulsion (NE) loaded with an in vitro optimized bactericidal essential oil blend of 50% oregano, 40% thyme, and 10% lemongrass and to evaluate its potential at three different concentrations (0.5%, 1%, and 2%) in the inactivation of *Escherichia coli*, *Staphylococcus aureus*, and *Salmonella enterica* serotype Enteritidis inoculated in rainbow trout fillets stored at 4 °C for 9 days. Regarding the NE, the nanometric size (<100 nm) with low polydispersion (0.17 ± 0.02) was successfully obtained through ultrasound at 2.09 W/cm^2^. Considering the three concentrations used, *S*. Enteritidis was the most susceptible. On the other hand, comparing the concentrations used, the NE at 2% showed better activity, reducing *S.* Enteritidis, *E. coli*, and *S. aureus* by 0.33, 0.20, and 0.73 log CFU/g, respectively, in the trout fillets. Thus, this data indicates that this is a promising eco-friendly alternative to produce safe fish for consumption and reduce public health risks.

## 1. Introduction

Mixture design (MD) is a quality technology that allows for achieving excellence in a product [1]. Furthermore, the optimization of essential oil blends (EOBs) through MD is an emerging approach that allows for maximizing antimicrobial activity [2,3], including the simultaneous inactivation of different pathogens [4]. For these purposes, obtaining EOB through MD may increase the mixture quality at a low cost, which can be a powerful incentive to enable the use of the synergistic or additive potential of EOs by food industries [2,3,4,5]. However, studies with this approach are limited to in vitro assays, and there is only one report evaluating the blend’s effectiveness in any food matrix in the literature [6]. Our research group is at the forefront of investigating and implementing the in situ application of EOB with MD [4,6]. Despite that, further research is warranted to address this significant gap in the literature by comprehensively exploring the potential of optimized EOB for oxidative and antimicrobial control in food products.

Studies utilizing MD to obtain EOB have successfully achieved mixtures capable of simultaneously inactivating in vitro *Bacillus subtilis*, *Staphylococcus aureus*, and *Escherichia coli* as well as *Candida tropicalis* [3,7]. Alternatively, these studies have demonstrated the individual efficacy against *Pseudomonas aeruginosa*, *E. coli*, and *S. aureus* [8,9]. Recently, our research group achieved a bactericidal EOB with oregano (ORE; *Origanum vulgare*), thyme (THY; *Thymus vulgaris*), and lemongrass (LG; *Cymbopogon citratus*) optimized for simultaneous inactivation in vitro through the minimum bactericidal concentration (MBC) of *Salmonella enterica* serotype Enteritidis, *Escherichia coli*, and *Staphylococcus aureus* [4]. Nevertheless, the subsequent essential step toward comprehending the efficacy of this formulated EOB entails its application within the food matrix. This is imperative considering that these three bacteria are accountable for numerous foodborne outbreaks globally, which transpire at various food production and transportation junctures, chiefly along the cold chain [9].

Considering the growing global demand for fish and the public health risks from its consumption, the Food and Agriculture Organization has encouraged studies of conservation methods to ensure the continuous supply of safe and high-quality fish, meeting the Sustainable Development Goal (SDG), which aims to boost the fish production chain in a sustainable way [10]. Therefore, secure natural alternatives using eco-friendly technologies for food applications is one of today’s top trending topics [11]. Indeed, fish was associated with 37 outbreaks reported by 50 US states in addition to Washington, D.C., and Puerto Rico in 2017 [12], in addition to 30 outbreaks in European Union Member States (EU MSs) in 2021 [13].

In this context, over recent years, using EOs as a strategy for food quality control has proved to be a promising approach, mainly to be considered an effective, natural, and eco-friendly plant-based alternative against pathogens [9,14]. In this sense, different strategies to try to improve the safety, quality, and shelf life have been evaluated with individual EOs in fish, such as the use of thyme or garlic essential oil (EO) together with vacuum packaging in hot smoked rainbow trout [15], oregano EO with chitosan in the storage of red porgy (*Pagrus pagrus*) [16], turbot (*Scophthalmus maximus*) fillets treated with clove, cumin, or spearmint EO fumigation [17], the use of bio-nanocomposite active packaging films with carboxymethyl cellulose, myrrh gum, TiO_2_ nanoparticles, and dill essential oil for preserving fresh fish (*Cyprinus carpio*) [18], a whey protein coating containing nanoliposome dill (*Anethum graveolens* L.) essential oil [19], pomelo peel essential oil applied in varieties of freshwater fish (Rohu, Bahu, Silver carp) [20], and grape and cinnamon EO NEs applied to chilled flathead mullet fillets [21]. Additionally, an oil-in-water emulsion with a mixture of these three EOs (ORE, THY, and LG) controlled oxidation and extended the shelf life of trout fillets [6]. However, despite several advantages that the development of EOBs present [4,6], currently, the evaluation of optimized antimicrobial EOBs in fish storage is still unexplored, yet the assessment of these blends in the control of pathogens in trout rainbow fillets could represent an essential response to the demands of FAO and the United Nations [22,23].

The ORE, THY, and LG EOs have broad antimicrobial action, which has already been documented in the literature [24,25]. Similarly, the combination of aldehydes and phenols has proven effective against Gram-positive and Gram-negative bacteria [4,26,27]. Moreover, the genus of these EOs holds significant interest due to their antioxidant and antimicrobial activities, with research conducted in numerous countries globally [28] underscoring their scientific and economic importance (forecasted to reach a value of USD 15.3 billion by 2027; Ref. [29]). For these reasons, combining these three EOs has considerable potential as an alternative antimicrobial for food products. At the same time, an encapsulation system can enhance their effectiveness or preserve their original biological activities [30], further expanding their applicability and utility in food preservation.

Ultrasound represents a high-energy technique harnessing acoustic cavitation to induce pressure fluctuations, leading to localized turbulence that facilitates droplet rupture and size reduction, ultimately yielding promising nanoemulsions (NEs) [30,31,32]. This method enables the production of NEs characterized by nanometric dimension and uniform droplet distribution [30]. Additionally, numerous studies have demonstrated the successful fabrication of NEs loaded with EOs or EOB, exhibiting optimized in vitro physicochemical [32,33] and biological [30] properties. Furthermore, prior studies developed by our group indicated that size reduction in NEs enhances the bioactivity of EOs compared to their free forms [30,34], in addition to safeguarding the EOs due to their high sensitivity to processing and storage conditions, such as heat, temperature, the presence of oxygen, and the macronutrients found in food matrices [35]. In this sense, nanoencapsulation protects and improves the efficiency and integrity of the biological activity of EOs [30]. However, the in situ evaluation of NEs loaded with optimized EOBs represents a crucial gap in the existing literature, especially in fish.

These facts informed the aim of this study, which was to obtain and characterize an oil-in-water nanoemulsion loaded with an optimized bactericidal blend of 50% ORE, 40% THY, and 10% LG and to evaluate its potential at three different concentrations (0.5%, 1%, and 2%) in the inactivation of *S*. Enteritidis, *E. coli*, and *S. aureus* in trout (*Oncorhynchus mykiss*) fillets stored at 4 °C for 9 days.

## 2. Material and Methods

### 2.1. Material

The ORE, LG, and THY EOs were acquired from Quinari (Ponta Grossa, Brazil). The Tween 80 was purchased from Rei-Sol (Rio de Janeiro, Brazil). The composition of the EOs was previously characterized and quantified through gas chromatography coupled to mass spectrometry (GC-MS) and flame ionization detector (FID) [4].

### 2.2. Nanoemulsion Preparation

For emulsion preparation, the bactericidal EOB with ORE, THY, and LG (50:40:10, respectively) was previously validated in vitro by Torres Neto et al. [4] and was used as the oil phase. The aqueous phase consisted of Tween 80 (ratio 2:1 with oil phase; Ref. [36]) in ultrapure water (Mili-Q IQ 7005, Merck, Darmstadt, Germany). Both phases were mixed and homogenized through an Ultraturrax (IKA T10, Staufen, Germany) for 10 min at 13,400 rpm, reaching a final EO blend concentration of 2% (*w*/*v*). For drop size reduction, the emulsion was sonicated with ultrasound (VC-750 Ultrasonic Processor, 20 kHz, 750 W, Vantaa, Finland) equipped with a 19 mm diameter probe (Sonics, Materials Inc., Newtown, PA, USA). The ultrasound configuration was adjusted according to Jiménez et al. [37], with slight modifications, in which it used 30% amplitude for 10 min in an ice bath for temperature control. In short, the effective power was 2.09 W/cm^2^, and the acoustic density energy (AED) was 12.56 kJ/mL [30,36]. After preparation, the nanoemulsion was subjected to characterization (Section 2.3).

### 2.3. Nanoemulsion Characterization

#### 2.3.1. Drop Size, Polydispersity Index, and Zeta Potential (ζ-Potential)

The droplet size distribution was determined using the Dynamic Light Scattering (DLS) method, and the ζ-potential measurements were performed through a Zetasizer Nano (Model 590, Malvern Instruments, UK). The hydrodynamic mean diameter (z-average) and polydispersity index (PDI) were determined. Furthermore, the size distribution was expressed with an adaptation of the radar charts [30,38], wherein the D10, D50, and D90 values (representing the size of 10%, 50%, and 90% of the drop population, respectively) of cumulative intensity (D*i*), volume (D*v*), and number (D*n*) were utilized.

#### 2.3.2. Confocal Laser Scanning Microscopy (CLSM)

The CLSM was conducted using a 65× objective lens to capture images of the sample. The emulsion (30 μL) was placed on a glass slide, labeled with Nile Red, and subsequently examined using CLSM (excitation wavelength: 543 nm, emission wavelength: 605 nm) [39,40] with a Leica TCS SP5 system (Leica Microsystems GmbH, Heidelberg, Germany).

### 2.4. Antibacterial Activity

#### 2.4.1. Bacterial Strain and Culture Conditions

The *E. coli* ATCC 25922, *S.* Enteritidis ATCC 13076, and *S. aureus* ATCC 14458 were obtained from the Oswaldo Cruz Foundation (FIOCRUZ, Rio de Janeiro, Brazil). The strains were stored in methylene blue eosin (EMB; Kasvi, Madrid, Spain), Xylose Lysine Deoxycholate (XLD; Kasvi, Spain), and tellurite–egg yolk agar (Baird-Parker Agar (BPA); Kasvi, Spain) (Sigma-Aldrich, Darmstadt, Germany), respectively. For the inoculum, five characteristic colonies of each strain were selected to be reactivated in individual tubes containing 50 mL of Brain Heart Infusion (Kasvi, Spain) at 37 °C/18–24 h, resulting in a final concentration of 8 log CFU/mL for each bacterium.

#### 2.4.2. Trout Fillet Preparation

Fresh, skinned fillets sourced from farmed rainbow trout (*Oncorhynchus mykiss*) were procured from a local fish farm in Rio de Janeiro, Brazil, and promptly transported to the laboratory within an ice chest box to maintain their freshness and integrity. Each fillet, weighing 50 g, was subjected to random allocation into one of four distinct treatment groups: control, NE0.5, NE1.0, and NE2.0. These treatments corresponded to the absence of EOB and the presence of 0.5%, 1.0%, and 2.0% (*w*/*v*) of the optimized blend, equating to concentrations of 0.3, 0.6, and 1.2 mg/g of fillet, respectively. For simulating post-cross-contamination scenarios, the NEs were uniformly sprayed onto the fillets (±3 mL) and allowed to dry at room temperature within a Dryflow cabin for 2 min. Subsequently, 500 μL of inoculum of each bacterial strain was individually spread onto the trout fillets (Section 2.4.1), allowing for a 5 min adherence period within a bacteriological cabin. Following this, the fillets were individually vacuum-packed using nylon/polyethylene bags employing a CV65 model (Conceito Vácuo, São Paulo, Brazil). These vacuum-packed fillets were then stored at a temperature of 4 ± 1 °C and subjected to analysis for each bacterial strain on days 0, 3, 6, and 9, following the methodology outlined in the prior investigation conducted by Monteiro et al. [41]. All the experiments were performed under sterile conditions in triplicate (*n* = 3).

#### 2.4.3. Microbiological Analysis

The sample aliquots (10 g) were homogenized in 90 mL of saline solution (0.85%, *w*/*v*) through the stomacher for 2 min. Subsequently, serial decimal dilutions ranging from 10^−2^ to 10^−5^ were meticulously prepared in saline solution (0.85% *w*/*v*) and subsequently streaked onto plates containing specific culture media tailored for each strain, as detailed in Section 2.4.1. The plates were then incubated at 37 °C for 18 to 24 h for the EMB and tellurite–egg yolk agar in BPA, and at 37 °C for 24 to 48 h for the XLD. All the experimental procedures were meticulously conducted in triplicate.

### 2.5. Statistical Analysis

The bacterial growth parameters (lag phase and µmax) were obtained through the DMFit program version 2.0. (Institute of Food Research, Norwich, UK) by the primary predictive model [42], and the differences among treatments were identified by one-way ANOVA with Tukey’s post hoc test (*p* < 0.05) using XLSTAT software, version 2021.1 (Addinsoft, New York, NY, USA). This was also used for Pearson’s correlation test to identify associations between the nanoemulsion characteristics and their antibacterial effects.

## 3. Results

### 3.1. Drop Size and Distribution

**Figure 1:**
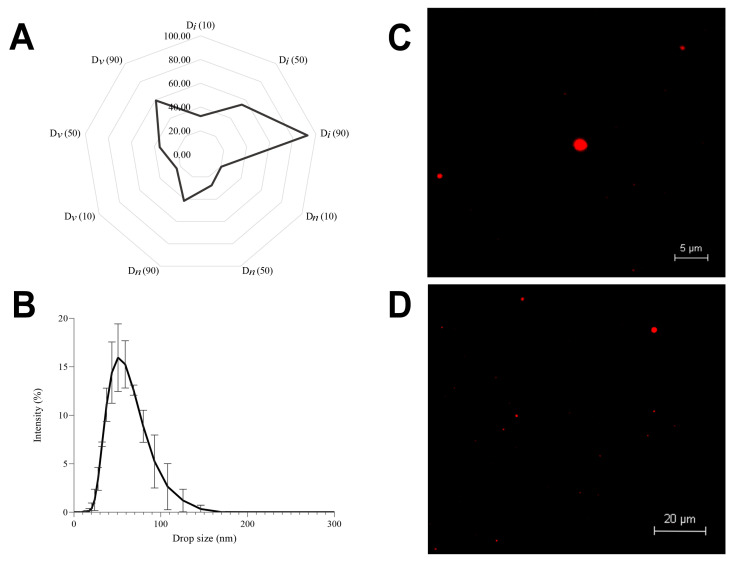
Radar chart demonstrating D_10_, D_50_, and D_90_ values (size of 10%, 50%, and 90% of the drops population) of cumulative intensity (D*i*), volume (D*v*), and number (D*n*) (**A**); droplet size profile of cumulative intensity of the nanoemulsion with 2% essential oil (EO) blend (**B**); confocal microscopy images of the nanoemulsion with 2% EO blend at 5 µm (**C**) and 20 µm (**D**) resolutions. The size data are expressed in nanometers (*n* = 3).

**Figure 2:**
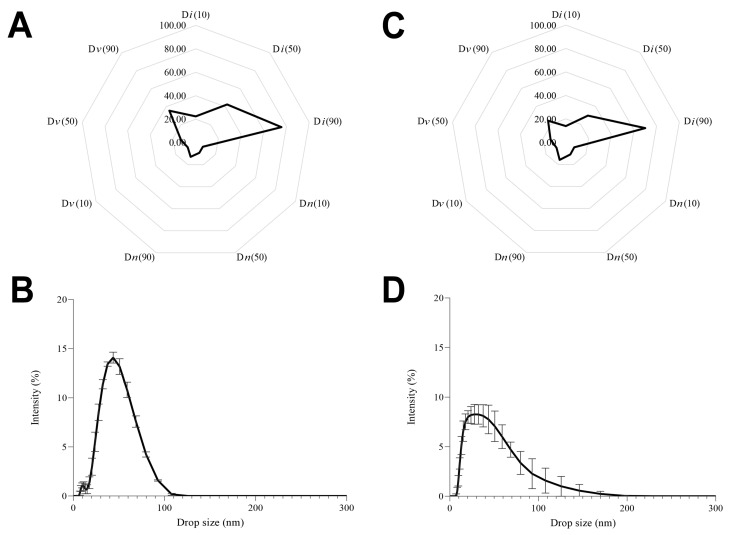
Radar chart demonstrating D10, D50, and D90 values (size of 10%, 50%, and 90% of the drops population) of cumulative intensity (D*i*), volume (D*v*), and number (D*n*) of the nanoemulsion with 1% essential oil (EO) blend (**A**) and 0.5% EO blend (**C**); droplet size profile of cumulative intensity of the nanoemulsion with 1% EO blend (**B**) and 0.5% EO blend (**D**). The size data are expressed in nanometers (*n* = 3).

All percentages of drop diameters obtained using intensity distribution (D*i*) were on the nanoscale below 100 nm (Figure 1A and Figure 2). Furthermore, (D*v* (90)) and (D*n* (90)) exhibited droplet sizes ≤ 60 nm and <50 nm, respectively (Figure 1A and Figure 2). Through the radar chart and CLSM, we can infer that the formulation was predominantly nanometric. Despite the presence of larger droplets, it was observed that a few big droplets (Figure 1C,D) were below 100 nm (D*i* (90); Figure 1A), and most of the droplet sizes were close to 50 nm (D*n* (90); Figure 1A), inferring low polydispersity. The *z*-average and PDI were 50.21 ± 0.63 nm and 0.17 ± 0.02, respectively, thereby confirming the homogeneity of the nanometric system (polydispersity < 0.25) [43].

### 3.2. Antibacterial Activity of Nanoemulsions in Trout Fillets

Any treatment immediately reduced *E. coli*, *S.* Enteritidis, and *S. aureus*, which had an average initial count of 6.91, 6.67, and 6.45 log CFU/g, respectively. No lag phase was observed for *E. coli* and *S*. Enteritidis in any treatment (Table 1), and NE_2_ (1.2 mg/g of fillet) was the only one increasing the lag phase for *S. aureus* (*p* < 0.05). Regarding µmax, the NE decreased it in all strains, wherein *S.* Enteritidis showed more susceptibility at the three concentrations tested, followed by *E. coli* and *S. aureus*. Within the NE treatments, NE_2_ was the only one decreasing the growth rate of the three bacterial strains (Table 1), resulting in the highest reductions by 0.73, 0.33, and 0.20 log CFU/g for *S. aureus*, *S*. Enteritidis, and *E. coli* compared with the control, respectively, at the end of the storage (Table S1). It is worth emphasizing the efficiency of the NE_2_ since its effect was observed in the worst scenario (high initial bacterial counts). Furthermore, marginal significant correlations were observed between droplet sizes and growth control of *S*. Enteritidis, *E. coli*, and *S. aureus* (Table 2). Considering 90% of the drop population, D*i*, D*v*, and D*n* had marginally negative correlations with *S.* Enteritidis reduction, as did D*v* and D*i* with *E. coli*. Otherwise, the three parameters representing the drop population exhibited marginal positive correlations with *S. aureus* reduction over refrigerated storage.

## 4. Discussion

### 4.1. Drop Size and Distribution

The distribution intensity (D*i*) emphasizes the larger drops in the nanoemulsion through the Brownian motion and Stoke–Einstein relationship. Moreover, the volume (D*v*) and number (D*n*) distribution, in turn, are obtained through D*i* by the Zetasizer software version 7.13 [44], where D*v* expresses the predominant volume in the distribution of the drops and D*n* the predominant size, emphasizing the smaller drops present in our nanoemulsion. In short, these three parameters are different representations of the same population of drops [45]. Moreover, the CLSM is an attractive complementary analysis to DLS [46], making it possible to comprehend the droplet distribution’s size profile better.

The small size and monomodal distribution (Figure 1A,B) observed can be attributed to ultrasound treatment associated with Tween 80. The shockwaves provoked by ultrasonic cavitation break up and intermingle the oil and water phases, converting large droplets into smaller ones [41,42]. The polysorbate 80 can rapidly adsorb to the droplet surfaces and reduce the interfacial tension [43]. Various factors influence the droplet size of NEs produced via the ultrasound method. Firstly, extended processing time may result in overprocessing, leading to droplet coalescence and subsequent size enlargement. Secondly, the ratio of EOB to Tween 80 is significant, as excess surfactant can promote the formation of larger micelles post-ultrasound treatment. Lastly, inadequate surfactant concentration relative to the oil content can also increase droplet size [30]. In the current study, the formulated NE displayed nanometric and uniformly sized droplets, suggesting mitigation of the previously mentioned effects. This observation can be ascribed to the specific EOB to Tween 80 ratio and the ultrasound parameters applied. Additionally, the “fingerprint” of the formulation, as depicted in the radar chart (Figure 1A,B), exhibited a characteristic of uniform monomodal profile distribution, which aligns with findings from prior research studies.

Hasheminya and Dehghannya [47] used Tween 80 and span 80 surfactants with high-intensity ultrasound (encapsulated probe with 7mm diameter and 100 mm length at 400 W power, 24 kHz frequency, and 100% amplitude at 25 °C for 20 min) with the *Froriepia subpinnata* (Ledeb.) baill essential oil. The authors achieved an average droplet size (*z*-average) of 84.32 nm in a 1:1 ratio of surfactant and EO. Torres Neto et al. [30], employing a combination of essential oil blend (EOB) consisting of oregano EO and lemongrass EO with Tween 80 at ratios of 1:1 and 1:2, achieved Di 90 values ranging from 20.63 ± 3.61 to 168.9 ± 30.78 nm. Notably, the highest Di 90 values (<100 nm) were observed with a 1:1 ratio of EOB and Tween 80. Additionally, similar to the current study, a trend towards smaller droplet sizes was noted with an increase in Tween 80 concentration relative to EOB. Da Silva et al. [36], utilizing 157.5 W for 4.9 min, obtained mean droplet sizes (Di 50) ranging from 54.47 to 84.07 nm (oregano EO, carvacrol, or thymol, and Tween 80). Yang et al. [33], employing 350 W for durations of 5 and 15 min, achieved sizes between 16.3 and 17 nm (thyme EO and Tween 80). Other coatings were utilized to obtain nanoemulsions (NE) through the ultrasound method, such as alginate and Tween 80 (in a 1:1:1 ratio), resulting in coating lemongrass EO with sizes ranging from 34.95 to 5.12 nm [48].

Furthermore, the NE showed a zeta potential slightly negative and close to zero (−6.90 ± 0.68 mV) being justified by the surfactant used, which attributes the surface charge in the NE [49]. The Tween 80 is a non-ionic surfactant [50]; however, when Tween 80 is exposed to the ultrasound process, some residues are produced (free fatty acids), making the NE surface charge negative [50]. Similarly, Da Silva et al. [36] used Tween 80 and oregano EO, carvacrol, or thymol at ratios of 1:1 and 1:3.5. These formulations achieved the highest charge at −12.40 ± 0.72 mV with 412 W for 10 min. Furthermore, Hemmatkhah et al. [51] coated cumin seed EO with Tween 80, showing a zeta-value of  − 0.3 mV using ultrasound at 200 W for 15 min. Moreover, Torres Neto et al. [30], using EOB (oregano EO and lemongrass EO) with Tween 80 (1:1 and 1:2), achieved zeta values ranging from −4.45 ± 0.74 at −8.07 ± 0.79 mV with 300 W for 18 min.

### 4.2. Antibacterial Activity of Nanoemulsions in Trout Fillet

The absence of the lag phase (Table 1) can be explained by two main factors: the increase in water activity conferred by the NE addition [52], permitting greater initial access to nutrients, and the delayed release of the EO blend by the NE [53], which contributed to no bacterial adaptation period. On the other hand, the action of the NEs was better observed in the exponential phase (µmax) and the treatment at higher EOB concentration (Table 1). All our formulations had a uniform droplet size below 100 nm (Section 3.1). Indeed, these characteristics are related to an increase in passive transport through the cell membrane, fusion with the phospholipid bilayer [54], including a lower loss of EO blend due to absorption by high-fat muscle from trout, protecting the EO, and allowing a higher delivery to the bacterial cell throughout refrigerated storage.

Marginal significant correlations (Table 2) reinforce our findings concerning the sensibility order of the bacterial strains to nanoemulsions of the EOB evaluated, in which *S.* Enteritidis was more susceptible at the three concentrations tested, followed by *E. coli* and *S. aureus,* further making the relationship between droplet size and EO concentration perceptible, which was also observed in the radar charts (Figure 1A and Figure 2A,C). For *S*. Enteritidis and *E. coli*, the smaller the drop size, the higher the antibacterial activity of the EOB nanoemulsion (Table 2), effective at 0.5% and 1%, respectively (Table 1). On the other hand, the larger the drop size, the higher the antibacterial activity of EOB nanoemulsion against *S. aureus*, possibly due to its higher resistance to EOB and needing higher concentrations than 1% to be effective (Table 1), which was also observed in our previous in vitro study (Torres Neto et al. [4]).

Currently, few studies have evaluated the antimicrobial effect of oil-in-water nanoemulsions with EOs against *E. coli*, *S. aureus*, or *S*. Enteritidis inoculated in fish, and the existing ones have a high variation in EO species, type of coating, inoculated load, storage period, and food matrix evaluated [55]. In the study of Raji et al. [56], *E. coli* O_157_:H_7_ was inoculated (10^6^ CFU/g) into trout fillets coated with chitosan nanoemulsions with *Zataria multiflora* or *Bunium persicum* EOs, which, after 12 days, allowed a reduction of 0.59 and 0.35 log CFU/g at 0.5%, and 0.44 and 0.44 log CFU/g at 1% of EOs, respectively. The same was observed for chitosan nanoemulsion with *Bunium persicum* EO (0.5%), reducing *E. coli* O_157_:H_7_ growth by 1.22 log CFU/g in trout fillets after 8 days of refrigerated storage [57]. However, it is essential to emphasize that chitosan without EOs was effective in both previous studies, decreasing *E. coli* growth by 0.29 and 0.85 log CFU/g, respectively, and contributing additively to the EOs’ activity. Stratakos and Grant [58] obtained a nanoemulsion similar to the present study, stabilized with polysorbate 80 and drop sizes between 100 and 60 nm. These authors inoculated *E. coli* (5 log_10_ CFU/g) in beef samples and reported a reduction of 0.23 and 0.37 log CFU/g by washing with thyme and carvacrol nanoemulsions (0.8%) after 7 days at 4 °C, respectively. Torres Neto et al. [6] presented the first report on controlling psychotropic bacteria from the blend of ORE and LG in trout fillets, increasing the shelf life by 13 h.

Based on the literature data and the high initial load of the bacterial strains used in this study, the nanoemulsions containing optimized ORE, THY, and LG EOB showed a promising antibacterial effect in refrigerated stored trout fillets. In other words, our formulation showed bacterial reductions close to other studies, meaning a potential finding since the studies used isolated EOs to specific bacteria instead of optimized EOB for simultaneous bacterial activity against three different bacteria, which could have decreased the efficiency of the EOs. In short, the composition of the EOs consisted of ORE rich in carvacrol (70.3%), p-cymene (10.4%), γ-terpinene (4.8%), (E)-β-caryophyllene (4.7%), linalool (2.2%), and myrcene (1.6%); THY in thymol (31.2%), carvacrol (25.5%), p-cymene (21.7%), linalool (6%), limonene (3.4%) and borneol (3.4%); and LG with geranial (45.5%), neral (33.7%), geraniol (4.5%), geranyl acetate (1.5%), citronellal (1.3%), and citronellol (1.1%) [4].

Additionally, the broadened scope of antibacterial activity against the three distinct bacteria can be rationalized by the amalgamation observed in the functional groups existing in the EOs, such as phenols (like carvacrol and thymol) and aldehydes (such as geranial and neral), which serve to enhance activity owing to their status as two of the most potent antimicrobial functional groups [27]. Furthermore, hydrocarbons play a pivotal role, augmenting the membrane permeability of other compounds [59,60]. Moreover, it is noteworthy that the requisite in situ concentrations generally range from 10 to 100 times higher than those in vitro [61]. However, in our investigation, this discrepancy was less than fourfold, thus underscoring the significant antibacterial efficacy of the combination of NE and optimized EOB. It is also pertinent to mention that the concentrations employed in our study were either below or equivalent to the maximum permissible concentration of free EO in meat, as deemed acceptable by consumers (0.1% *v*/*w*; 1 mg/g), as elucidated by Possas et al. [62].

## 5. Conclusions

Considering oil-in-water nanoemulsion, the nanometric size (<100 nm) with a monodisperse formulation was successfully obtained through ultrasound at 2.09 W/cm^2^. *S.* Enteritidis showed more susceptibility at the three concentrations tested (0.5%, 1%, and 2%), followed by *E. coli* and *S. aureus*. The nanoemulsion with 2% optimized EOB showed better antibacterial activity against *S*. Enteritidis, *E. coli*, and *S. aureus*, with a reduction of 0.33, 0.20, and 0.73 log CFU/g in trout fillets after 9 days, respectively. This indicates promising results regarding controlling these three relevant foodborne pathogens in refrigerated stored trout. Lastly, this is the first in situ study evaluating the efficiency of a bactericidal-optimized EOB nanoemulsion. Therefore, this study lays the groundwork for forthcoming research initiatives focused on integrating this EOB nanoemulsion with various coatings (e.g., chitosan, alginate, proteins, polysaccharides, etc.), as well as non-thermal techniques (e.g., UV-C LED, ultrasound, high hydrostatic pressure, etc.). Furthermore, it encourages the exploration of optimization studies into different concentrations and sensory attributes considering varying temperatures, packaging methods, and storage periods, as well as exploring its combination with other emerging approaches (e.g., UV-C LED, ultrasound, high hydrostatic pressure), broadening the scope for potential applications and advancements in food preservation techniques.

## Figures and Tables

**Table 1:** Bacterial growth parameters of rainbow trout (*Oncorhynchus mykiss*) fillets coated with nanoemulsion with optimized essential oil (EO) blend ^€^ and stored at 4 ± 1 °C for 9 days.

	*Escherichia coli*		*Staphylococcus aureus*		*Salmonella* Enteritidis	
Treatments *	Lag Phase ^#^	µmax ^#^	Lag Phase ^#^	µmax ^#^	Lag Phase ^#^	µmax ^#^
Control	-	0.035 ± 0.01 ^a^	5.434 ± 0.34 ^a^	0.368 ± 0.03 ^a^	-	0.071 ± 0.00 ^a^
NE_0.5_		0.022 ± 0.00 ^a^	6.043 ± 0.33 ^a^	0.304 ± 0.03 ^a^		0.031 ± 0.00 ^b^
NE_1_		−0.022 ± 0.01 ^b^	6.159 ± 0.24 ^a^	0.298 ± 0.01 ^a^		0.025 ± 0.00 ^b^
NE_2_		−0.040 ± 0.01 ^b^	0.000 ± 0.00 ^b^	0.057 ± 0.01 ^b^		−0.018 ± 0.00 ^c^

Results are expressed as the mean ± standard deviation (*n* = 3). ^#^ Lag phase in days; µmax (exponential growth rate) in log CFU/g/h. * Control (absence of antioxidant); NE_0.5_ (0.5% of the optimized blend nanoemulsion); NE_1_ (1% of the optimized blend nanoemulsion); and NE_2_ (2% of the optimized blend nanoemulsion). ^€^ Optimized EO blend was composed of oregano (*Origanum vulgare*), thyme (*Thymus vulgaris*), and lemongrass (*Cymbopogon citratus*) at 50%, 40%, and 10%, respectively. Different letters indicate significant differences (*p* < 0.05).

**Table 2:** Pearson correlation between size parameters of the nanoemulsion (NE_0.5%_, NE_1%_, and NE_2%_) and bacterial reductions (final day–initial day) in rainbow trout (*Oncorhynchus mykiss*) during storage at 4 ± 1 °C for 9 days.

Variables ^#^	*Salmonella* Enteritidis	*Escherichia coli*	*Staphylococcus aureus*
D*i* (10)	−0.996	−0.872	0.877
D*i* (50)	−0.999 *	−0.896	0.844
D*i* (90)	−0.946	−0.733	0.972
D*n* (10)	−0.791	−0.475	0.763
D*n* (50)	−0.789	−0.472	0.761
D*n* (90)	−0.787	−0.470	0.759
D*v* (10)	−0.798	−0.484	0.770
D*v* (50)	−0.829	−0.532	0.805
D*v* (90)	−0.970	−0.787	0.975

* *p* < 0.05. ^#^ D10, D50, and D90 values (size of 10%, 50%, and 90% of the drop population) of cumulative intensity (D*i*), volume (D*v*), and number (D*n*) of the nanoemulsions: NE_0.5_ (0.5% of the optimized blend nanoemulsion); NE_1_ (1% of the optimized blend nanoemulsion); NE_2_ (2% of the optimized blend nanoemulsion). The optimized essential oil blend was composed of oregano (*Origanum vulgare*), thyme (*Thymus vulgaris*), and lemongrass (*Cymbopogon citratus*) at 50%, 40%, and 10%, respectively.

## Data Availability

The original contributions presented in the study are included in the article/Supplementary Materials; further inquiries can be directed to the corresponding author.

## Supplementary Materials

The following supporting information can be downloaded at: https://www.mdpi.com/article/10.3390/foods13101569/s1, Table S1: Bacterial count of rainbow trout (*Oncorhynchus mykiss*) fillets coated with nanoemulsions containing optimized essential oil (EO) blend^€^ and stored at 4 ± 1 °C for 9 days.

## References

[1] Orives J.R., Galvan D., Coppo R.L., Rodrigues C.H.F., Angilelli K.G., Borsato D. (2014). Multiresponse Optimisation on Biodiesel Obtained through a Ternary Mixture of Vegetable Oil and Animal Fat: Simplex-Centroid Mixture Design Application. Energy Convers. Manag..

[2] Fadil M., Fikri-Benbrahim K., Rachiq S., Ihssane B., Lebrazi S., Chraibi M., Haloui T., Farah A. (2018). Combined Treatment of *Thymus vulgaris* L., *Rosmarinus officinalis* L. and *Myrtus communis* L. Essential Oils against *Salmonella typhimurium*: Optimization of Antibacterial Activity by Mixture Design Methodology. Eur. J. Pharm. Biopharm..

[3] Ouedrhiri W., Balouiri M., Bouhdid S., Moja S., Chahdi F.O., Taleb M., Greche H. (2016). Mixture Design of Origanum Compactum, *Origanum majorana* and *Thymus serpyllum* Essential Oils: Optimization of Their Antibacterial Effect. Ind. Crops Prod..

[4] Torres Neto L., Monteiro M.L.G., Machado M.A.M., Galvan D., Conte Junior C.A. (2022). An Optimization of Oregano, Thyme, and Lemongrass Essential Oil Blend to Simultaneous Inactivation of Relevant Foodborne Pathogens by Simplex–Centroid Mixture Design. Antibiotics.

[5] Baj T., Kowalska G., Kowalski R., Szymańska J., Kai G., Coutinho H.D.M., Sieniawska E. (2023). Synergistic Antioxidant Activity of Four—Component Mixture of Essential Oils: Basil, Cedarwood, Citronella and Thyme for the Use as Medicinal and Food Ingredient. Antioxidants.

[6] Torres Neto L., Monteiro M.L.G., da Silva B.D., Galvan D., Conte-Junior C.A. (2024). Oil-in-Water Emulsion Loaded with Optimized Antioxidant Blend Improved the Shelf-Life of Trout (*Oncorhynchus mykiss*) Fillets: A Study with Simplex-Centroid Design. Sci. Rep..

[7] Chraibi M., Fadil M., Farah A., Lebrazi S., Fikri-Benbrahim K. (2021). Antimicrobial Combined Action of *Mentha pulegium*, *Ormenis mixta* and *Mentha piperita* Essential Oils against *S. aureus*, *E. coli* and *C. tropicalis*: Application of Mixture Design Methodology. LWT.

[8] Soulaimani B., Abbad I., Varoni E., Iriti M., Mezrioui N.-E., Hassani L., Abbad A. (2022). Optimization of Antibacterial Activity of Essential Oil Mixture Obtained from Three Medicinal Plants: Evaluation of Synergism with Conventional Antibiotics and Nanoemulsion Effectiveness. S. Afr. J. Bot..

[9] Wang N., Wang Y., Bai L., Liao X., Liu D., Ding T. (2023). Advances in Strategies to Assure the Microbial Safety of Food-Associated Ice. J. Futur. Foods.

[10] Food and Agriculture Organization. https://www.fao.org/3/cc0461en/online/cc0461en.html.

[11] Baptista R.C., Horita C.N., Sant’Ana A.S. (2020). Natural Products with Preservative Properties for Enhancing the Microbiological Safety and Extending the Shelf-Life of Seafood: A Review. Food Res. Int..

[12] CDC Foodborne Disease Outbreak Surveillance System. https://www.cdc.gov/fdoss/annual-reports/2017-report-highlights.html.

[13] European Food Safety Authority, European Centre for Disease Prevention and Control (2022). The European Union One Health 2021 Zoonoses Report. EFSA J..

[14] Ribeiro A.M., Estevinho B.N., Rocha F. (2021). Preparation and Incorporation of Functional Ingredients in Edible Films and Coatings. Food Bioprocess Technol..

[15] Erkan N. (2012). The Effect of Thyme and Garlic Oil on the Preservation of Vacuum-Packaged Hot Smoked Rainbow Trout (*Oncorhynchus mykiss*). Food Bioprocess Technol..

[16] Vatavali K., Karakosta L., Nathanailides C., Georgantelis D., Kontominas M.G. (2013). Combined Effect of Chitosan and Oregano Essential Oil Dip on the Microbiological, Chemical, and Sensory Attributes of Red Porgy (*Pagrus pagrus*) Stored in Ice. Food Bioprocess Technol..

[17] Cai L., Cao A., Li T., Wu X., Xu Y., Li J. (2015). Effect of the Fumigating with Essential Oils on the Microbiological Characteristics and Quality Changes of Refrigerated Turbot (*Scophthalmus maximus*) Fillets. Food Bioprocess Technol..

[18] Eshaghi R., Mohsenzadeh M., Ayala-Zavala J.F. (2024). Bio-Nanocomposite Active Packaging Films Based on Carboxymethyl Cellulose, Myrrh Gum, TiO2 Nanoparticles and Dill Essential Oil for Preserving Fresh-Fish (*Cyprinus carpio*) Meat Quality. Int. J. Biol. Macromol..

[19] Azizi M., Jahanbin K., Shariatifar N. (2024). Evaluation of Whey Protein Coating Containing Nanoliposome Dill (*Anethum graveolens* L.) Essential Oil on Microbial, Physicochemical and Sensory Changes of Rainbow Trout Fish. Food Chem. X.

[20] Yumnam M., Marak P.R., Gupta A.K., Rather M.A., Mishra P. (2023). Effect of Pomelo Peel Essential Oil on the Storage Stability of a Few Selected Varieties of Freshwater Fish. J. Agric. Food Res..

[21] Ameur A., Bensid A., Ozogul F., Ucar Y., Durmus M., Kulawik P., Boudjenah-Haroun S. (2022). Application of Oil-in-water Nanoemulsions Based on Grape and Cinnamon Essential Oils for Shelf-life Extension of Chilled Flathead Mullet Fillets. J. Sci. Food Agric..

[22] Monteiro M.L.G., Rosário D.K.A., de Carvalho A.P.A., Conte-Junior C.A. (2021). Application of UV-C Light to Improve Safety and Overall Quality of Fish: A Systematic Review and Meta-Analysis. Trends Food Sci. Technol..

[23] UN The 17 Goals. https://sdgs.un.org/goals.

[24] Hossen M.A., Shimul I.M., Sameen D.E., Rasheed Z., Dai J., Li S., Qin W., Tang W., Chen M., Liu Y. (2024). Essential Oil–Loaded Biopolymeric Particles on Food Industry and Packaging: A Review. Int. J. Biol. Macromol..

[25] Donsì F., Ferrari G. (2016). Essential Oil Nanoemulsions as Antimicrobial Agents in Food. J. Biotechnol..

[26] Nowak A., Kalemba D., Krala L., Piotrowska M., Czyzowska A. (2012). The Effects of Thyme (*Thymus vulgaris*) and Rosemary (*Rosmarinus officinalis*) Essential Oils on *Brochothrix thermosphacta* and on the Shelf Life of Beef Packaged in High-Oxygen Modified Atmosphere. Food Microbiol..

[27] Kalemba D., Kunicka A. (2003). Antibacterial and Antifungal Properties of Essential Oils. Curr. Med. Chem..

[28] Galvan D., Effting L., Torres Neto L., Conte-Junior C.A. (2021). An Overview of Research of Essential Oils by Self-organizing Maps: A Novel Approach for Meta-analysis Study. Compr. Rev. Food Sci. Food Saf..

[29] Markets M. Essential Oil Market. https://www.marketsandmarkets.com/Market-Reports/essential-oil-market-119674487.html.

[30] Torres Neto L., Monteiro M.L.G., Mutz Y.d.S., Tonon R.V., Conte-Junior C.A. (2023). Nanoemulsification of Essential Oil Blend by Ultrasound: Optimization of Physicochemical, Antioxidant Properties, and Activity against *Escherichia coli*. Food Bioprocess Technol..

[31] Alves de Aguiar Bernardo Y., Kaic Alves do Rosario D., Adam Conte-Junior C. (2023). Ultrasound on Milk Decontamination: Potential and Limitations Against Foodborne Pathogens and Spoilage Bacteria. Food Rev. Int..

[32] da Silva B.D., do Rosário D.K.A., Conte-Junior C.A. (2022). Can droplet size influence antibacterial activity in ultrasound-prepared essential oil nanoemulsions?. Crit. Rev. Food Sci. Nutr..

[33] Yang Z., He Q., Ismail B.B., Hu Y., Guo M. (2022). Ultrasonication Induced Nano-Emulsification of Thyme Essential Oil: Optimization and Antibacterial Mechanism against *Escherichia coli*. Food Control.

[34] da Silva B.D., do Rosário D.K.A., Neto L.T., Lelis C.A., Conte-Junior C.A. (2023). Antioxidant, Antibacterial and Antibiofilm Activity of Nanoemulsion-Based Natural Compound Delivery Systems Compared with Non-Nanoemulsified Versions. Foods.

[35] Hąc-Wydro K., Flasiński M., Romańczuk K. (2017). Essential Oils as Food Eco-Preservatives: Model System Studies on the Effect of Temperature on Limonene Antibacterial Activity. Food Chem..

[36] da Silva B.D., do Rosário D.K.A., de Aguiar Bernardo Y.A., Conte-Junior C.A. (2023). Improvement of Physicochemical and Antibacterial Properties of Nanoemulsified *Origanum vulgare* Essential Oil Through Optimization of Ultrasound Processing Variables. Food Bioprocess Technol..

[37] Jiménez M., Domínguez J.A., Pascual-Pineda L.A., Azuara E., Beristain C.I. (2018). Elaboration and Characterization of O/W Cinnamon (*Cinnamomum zeylanicum*) and Black Pepper (*Piper nigrum*) Emulsions. Food Hydrocoll..

[38] Bianchin M.D., Külkamp-Guerreiro I.C., de Oliveira C.P., Contri R.V., Guterres S.S., Pohlmann A.R. (2015). Radar Charts Based on Particle Sizing as an Approach to Establish the Fingerprints of Polymeric Nanoparticles in Aqueous Formulations. J. Drug Deliv. Sci. Technol..

[39] Chung C., Koo C.K.W., Sher A., Fu J.-T.R., Rousset P., McClements D.J. (2019). Modulation of Caseinate-Stabilized Model Oil-in-Water Emulsions with Soy Lecithin. Food Res. Int..

[40] Sharif H.R., Abbas S., Majeed H., Safdar W., Shamoon M., Khan M.A., Shoaib M., Raza H., Haider J. (2017). Formulation, Characterization and Antimicrobial Properties of Black Cumin Essential Oil Nanoemulsions Stabilized by OSA Starch. J. Food Sci. Technol..

[41] Monteiro M.L.G., Torres Neto L., Mutz Y.d.S., da Silva C.R., Cardoso A.C.C., Conte-Junior C.A. (2024). Optimized UVC-LED Condition to Improve the Shelf Life of Vacuum-Packed Refrigerated Stored Rainbow Trout (*Oncorhynchus mykiss*) Fillets. Food Control.

[42] Baranyi J., Roberts T.A. (1994). A Dynamic Approach to Predicting Bacterial Growth in Food. Int. J. Food Microbiol..

[43] Alves M.P., Scarrone A.L., Santos M., Pohlmann A.R., Guterres S.S. (2007). Human Skin Penetration and Distribution of Nimesulide from Hydrophilic Gels Containing Nanocarriers. Int. J. Pharm..

[44] Nobbmann U. D90, D50, D10, and Span—For DLS?. https://www.materials-talks.com/d90-d50-d10-and-span-for-dls/.

[45] Malvern Intensity—Volume—Number. https://www.malvernpanalytical.com/en/learn/knowledge-center/technical-notes/tn101104intensityvolumenumber.

[46] Rashed M.M.A., Ghaleb A.D.S., Li J., Al-Hashedi S.A., Rehman A. (2021). Functional-Characteristics of *Zanthoxylum schinifolium* (Siebold & Zucc.) Essential Oil Nanoparticles. Ind. Crops Prod..

[47] Hasheminya S.-M., Dehghannya J. (2022). Development and Characterization of *Froriepia subpinnata* (Ledeb.) Baill Essential Oil and Its Nanoemulsion Using Ultrasound. Food Bioprocess Technol..

[48] Salvia-Trujillo L., Rojas-Graü A., Soliva-Fortuny R., Martín-Belloso O. (2013). Physicochemical Characterization of Lemongrass Essential Oil–Alginate Nanoemulsions: Effect of Ultrasound Processing Parameters. Food Bioprocess Technol..

[49] Raviadaran R., Chandran D., Shin L.H., Manickam S. (2018). Optimization of Palm Oil in Water Nano-Emulsion with Curcumin Using Microfluidizer and Response Surface Methodology. LWT.

[50] McClements D.J. (2021). Advances in Edible Nanoemulsions: Digestion, Bioavailability, and Potential Toxicity. Prog. Lipid Res..

[51] Hemmatkhah F., Zeynali F., Almasi H. (2020). Encapsulated Cumin Seed Essential Oil-Loaded Active Papers: Characterization and Evaluation of the Effect on Quality Attributes of Beef Hamburger. Food Bioprocess Technol..

[52] McDonald K., Sun D.-W. (1999). Predictive Food Microbiology for the Meat Industry: A Review. Int. J. Food Microbiol..

[53] da Silva B.D., do Rosário D.K.A., Weitz D.A., Conte-Junior C.A. (2022). Essential Oil Nanoemulsions: Properties, Development, and Application in Meat and Meat Products. Trends Food Sci. Technol..

[54] Prakash A., Baskaran R., Paramasivam N., Vadivel V. (2018). Essential Oil Based Nanoemulsions to Improve the Microbial Quality of Minimally Processed Fruits and Vegetables: A Review. Food Res. Int..

[55] Barzegar F., Nabizadeh S., Kamankesh M., Ghasemi J.B., Mohammadi A. (2023). Recent Advances in Natural Product-Based Nanoemulsions as Promising Substitutes for Hazardous Synthetic Food Additives: A New Revolution in Food Processing. Food Bioprocess Technol..

[56] Raji F., Khanzadi S., Hashemi M., Azizzadeh M. (2019). Effect of Chitosan Coating Nano-Emulsion Containing *Zataria multiflora* and *Bunium persicum* Essential Oils on *Escherichia coli* O157:H7 in Vacuum-Packed Rainbow Trout Fillet. J. Human Environ. Heal. Promot..

[57] Kazemeini H., Azizian A., Shahavi M.H. (2019). Effect of Chitosan Nano-Gel/Emulsion Containing *Bunium persicum* Essential Oil and Nisin as an Edible Biodegradable Coating on *Escherichia coli* O157:H7 in Rainbow Trout Fillet. J. Water Environ. Nanotechnol..

[58] Stratakos A.C., Grant I.R. (2018). Evaluation of the Efficacy of Multiple Physical, Biological and Natural Antimicrobial Interventions for Control of Pathogenic *Escherichia coli* on Beef. Food Microbiol..

[59] Nazzaro F., Fratianni F., De Martino L., Coppola R., De Feo V. (2013). Effect of Essential Oils on Pathogenic Bacteria. Pharmaceuticals.

[60] Tiwari B.K., Valdramidis V.P., O’ Donnell C.P., Muthukumarappan K., Bourke P., Cullen P.J. (2009). Application of Natural Antimicrobials for Food Preservation. J. Agric. Food Chem..

[61] da Silva B.D., Bernardes P.C., Pinheiro P.F., Fantuzzi E., Roberto C.D. (2021). Chemical Composition, Extraction Sources and Action Mechanisms of Essential Oils: Natural Preservative and Limitations of Use in Meat Products. Meat Sci..

[62] Possas A., Posada-Izquierdo G.D., Pérez-Rodríguez F., Valero A., García-Gimeno R.M., Duarte M.C.T. (2017). Application of Predictive Models to Assess the Influence of Thyme Essential Oil on *Salmonella* Enteritidis Behaviour during Shelf Life of Ready-to-Eat Turkey Products. Int. J. Food Microbiol..

