# Supplementary material for: Ultrasound-Assisted Nanoemulsion Loaded with Optimized Antibacterial Essential Oil Blend: A New Approach against Escherichia coli, Staphylococcus aureus, and Salmonella Enteritidis in Trout (Oncorhynchus mykiss) Fillets
Source: Foods. 2024 May 17;13(10):1569. doi: 10.3390/foods13101569 (PMC11120578; doi:10.3390/foods13101569)
Supplement: Supplementary file 1 [file foods-13-01569-s001.zip › foods-2981563-supplementary.pdf]

Supplementary Table S1. Bacterial count of rainbow trout (*Oncorhynchus mykiss*) fillets coated with nanoemulsions containing optimized essential oil (EO) blend[€] and stored at 4 ± 1°C for 9 days.

Escherichia coli				
Treatments*	Day 0	Day 3	Day 6	Day 9
Control	6.91 ± 0.38	6.80 ± 0.09	6.93 ± 0.11	6.98 ± 0.13
$NE_{0.5}$	6.97 ± 0.83	7.08 ± 0.16	7.02 ± 0.12	7.03 ± 0.06
NE_1	7.22 ± 0.47	6.94 ± 0.12	7.13 ± 0.12	6.90 ± 0.38
NE_2	7.23 ± 0.17	6.84 ± 0.08	6.85 ± 0.06	6.88 ± 0.31
Staphylococcus aureus				
Treatments	Day 0	Day 3	Day 6	Day 9
Control	6.45 ± 0.19	5.46 ± 0.08	6.18 ± 0.15	7.25 ± 0.04
$NE_{0.5}$	6.41 ± 0.07	6.05 ± 0.10	6.32 ± 0.09	7.13 ± 0.01
NE_1	6.23 ± 0.18	6.11 ± 0.07	6.20 ± 0.02	6.99 ± 0.04
NE_2	6.30 ± 0.16	5.95 ± 0.18	5.67 ± 1.02	7.03 ± 0.04
Salmonella Enteritidis				
Treatments	Day 0	Day 3	Day 6	Day 9
Control	6.67 ± 0.17	6.89 ± 0.04	6.97 ± 0.15	7.00 ± 0.19
$NE_{0.5}$	6.58 ± 0.18	6.94 ± 0.11	6.95 ± 0.11	6.88 ± 0.36
NE_1	6.69 ± 0.07	6.72 ± 0.16	6.91 ± 0.09	6.71 ± 0.30
NE_2	6.82 ± 0.11	6.65 ± 0.12	6.85 ± 0.09	6.60 ± 0.06

Results are expressed as the mean ± standard deviation ($n = 3$). *Control (absence of antioxidant); $NE_{0.5}$ (0.5% of the optimized blend nanoemulsion); NE_1 (1% of the optimized blend nanoemulsion); NE_2 (2% of the optimized blend nanoemulsion). [€]Optimized EO blend was composed of oregano (*Origanum vulgare*), thyme (*Thymus vulgaris*), and lemongrass (*Cymbopogon citratus*) at 50%, 40%, and 10%, respectively.
